# Telemedicine and the Doctor/Patient Relationship

**DOI:** 10.5935/abc.20190117

**Published:** 2019-07

**Authors:** Protásio Lemos da Luz

**Affiliations:** Universidade de São Paulo - Hospital das Clinicas Instituto do Coração, São Paulo, SP - Brazil

**Keywords:** Physician-Patient Relations, Telemedicine, Cost Saving, Medical Care, Humanism, Patient Participation

## Introduction

A.C., a friendly Italian man, received coronary stents 8 years ago and had been feeling well until 3 months ago. He then had progressive breathing difficulty with tightness in his chest. He had loss of appetite and lost 4 kg in that period, but continued to work with good disposition. He had been to two hospital visits, in which he was informed that his lung was normal, based on X-ray. They had not reached final diagnosis, so he came to the office. His general appearance was normal; no abnormality was found on visual inspection. He had normal vital signs; no pallor to suggest anemia. The lungs, heart, abdomen and lower limbs were normal on examination, and resting ECG revealed no abnormalities either. As I palpated his neck, I noticed a mass on the left side. Subsequent examinations proved that a tumor compressed the trachea, which explained his respiratory difficulty. This is a typical example of a case in which only complete clinical examination leads to diagnosis. Telemedicine (TM) would not allow this, as simple inspection did not give any clues, and the clinical history suggested several possibilities, such as heart disease, pulmonary disease, diabetic decompensation, uremia or anemia.

There is now a major debate, both at the health system and at the individual telemedicine level. Telecommunication technologies have long been used in teaching, in science, in the formation of groups for the study of diseases, in the transmission of diagnostic or therapeutic medical procedures, in imaging interpretation by specialists, in teleconference with undisputed efficiency, and will not be considered here. An interesting fact: many even said that, with the Internet, medical conferences would be emptied, because everything could be seen from a distance.

That simply did not happen - medical conferences continue to attract large audiences, as much as before the online broadcasts. This proves that man is a social being and likes to live with his peers.

### Telemedicine and the Doctor/Patient Relationship

Telemedicine here is considered the medical care for patients without face-to-face contact. An essential aspect is that telemedicine requires its own structures. It requires a central station that can receive information, equipped with medical professionals ready to respond; it also requires the other end, i.e., doctors or patients providing correct data for evaluation. Recent iPhones are fundamental; they allow the exchange of images, texts; etc. Other devices record ECG, heart rate and blood pressure. And we are not far from the day that laboratory parameters will be transmitted online from skin sensors. Therefore, telecommunication systems are not an important limitation for telemedicine. Another fundamental aspect is the impact on health systems, including efficiency, costs, satisfaction and adherence of patients and doctor. As the implementation of telemedicine is relatively recent, there are only a few studies on this topic. A Cochrane review,^1^ which included 93 studies and 22,047 patients, analyzed 36 studies of cardiovascular diseases, 21 of diabetes, 9 of respiratory conditions, 7 of mental health, 6 in which a specialist needed to be consulted, 3 urogenital conditions, 3 with neurological lesions, and other minor ones. Comparing telemedicine with usual care, they showed that there was no difference in overall mortality from heart failure, that hospital admissions were reduced by 64% in some studies, and increased by 60% in others. There was some evidence for improved quality of life and, in diabetes, lower glycated hemoglobin was found; greater reductions in LDL and blood pressure were also found. There were no differences between face-to-face healthcare and telemedicine in cases of mental health. Regarding costs and patient acceptance, the authors found that there was insufficient data for conclusions. The authors concluded that the efficiency of telemedicine depends on multiple factors, such as whether it is used to monitor chronic conditions and known patients or to facilitate access to diagnostic services. Worthy of note is that all studies refer to monitoring of known chronic conditions, rehabilitation training offerings, healthy life education, specialist consultations or cognitive therapy; that is, those would always refer to cases of known diagnoses rather than primary evaluations. Another analysis by Ekeland et al.^2^ included 80 studies and found that 21 of them considered effective telemedicine, 18 were promising, but still inconclusive, and the others had limited and inconsistent evidence. Bertoncello et al.^3^ analyzed the factors involved in the efficiency of telemedicine according to reports from 25 critically chosen reviews, comprising 15 years (2000 to 2015) of observations. Hypertension, diabetes, asthma, chronic obstructive pulmonary disease, heart failure and elderly care were included. This analysis considered several factors that may influence efficiency in telemedicine, such as the geographical location of patients, demographic characteristics and diseases (the so-called “targets”), intensity of intervention, patient perspectives and engagement, education, caregivers, organizational model and ethical and economic issues. Interestingly, none of the studies looked at all of the factors; 44% focused on the “targets” and 24% on intervention intensity; 16% analyzed the patients’ perspective. On the other hand, issues such as location, ethical issues, patient engagement or caregiver perspectives were the least addressed items. The researchers’ main conclusion was that there is not yet enough concrete data on the multiple factors that influence efficiency telemedicine, and that further studies are needed to fill knowledge gaps in this area.

A major discussion is the doctor/patient relationship (DPR) in telemedicine. Traditionally, medicine has been based on the individual relationship between doctor and patient.^4^ This relationship has multiple cultural influences. In Brazil, people are very affectionate and zealous of their family relationships and their friendships. This affection extends to the doctor, which makes us all more sensitive. In the Anglo-Saxon culture, personal relationships are more distant and “colder”. In practice, this means that brazilians like to have “their doctor,” rather than being treated by a stranger. The patient’s trust in his/her doctor is not acquired in an instant, but in prolonged coexistence, especially in situations of risk. Patients are quite protective of their privacy, and rightly so; no one will talk about sexual impotence, relationships with spouses, children or family to a device that can record the conversation and even post it on Facebook. Likewise, an executive is not expected to report that his/her stress is a result of unsuccessful business; if this is announced, the situation will only get worse. How is it possible to convey affection, understanding, commitment, compassion, human warmth at last - without looking into the patient’s eyes? Another significant point is body language. It is well known to psychologists that about 80% of messages one person conveys to others is not verbal;^5^ it is conveyed through body posture, voice intonation, the way the person looks, how they move their hands and arms, the way they sit, whether they smile or not, and whether their smile is spontaneous or not. In short, there are whole books dedicated to this topic.^6^ Also, when the jury meets to judge major crimes, witnesses and defendants are heard personally. The purpose of that is precisely for the jurors to assess the authenticity of the accounts. Doctor/patient contact has a similar meaning. Both the doctor evaluates the patient and the patient evaluates the doctor. It is questionable whether an image could replace that personal contact with the same precision.

In a more general view, how do you know if the doctor on the other end of the line has the authority to give an opinion on that case? Countless patients seek the opinion of renowned doctors, even after they have gone through a number of other doctor’s visits. This only emphasizes one of the basic principles on which medicine is based: trust. In addition, does telemedicine lessen medical error? According to J. Groopman^7^ - doctors make mistakes in 15% of the cases on average. That’s a high percentage! The impact that telemedicine will have on this is unknown; there is no data.

Another key aspect is compensation. There are no established criteria; this should be a reason for wide discussion, as there are several points to be considered, such as public healthcare, health insurance and private patients. In this particular case, many medical entities and providers should participate.

Another point to consider is who/which entities should establish the rules of telemedicine practice. Usually, the Federal Medical Council, medical societies, medical colleges and patient representatives should be heard. So far, patients have been systematically excluded from similar discussions. This needs to be fixed. After all, patients are the goal of medical actions, they take the risks and pay for healthcare. Besides, this is critical - in the case of telemedicine, as in all medical procedures, the patient must explicitly agree with the process, as it involves potential privacy and confidentiality issues. Along these lines, patients’ rights to privacy, alternatives, potential risks and benefits must be preserved. As previously said, the patient does not always accept new technologies like the telemedicine. It is necessary to be clear that they have freedom of choice.

Therefore, which stance should we take on the current medical practice? Conceptually speaking, it is clear that telemedicine is here to stay. It is simply a matter of adapting it to medical practice. Having said this, I believe that:

- the first visit must be in person; neither anamnesis nor physical examination can be eliminated - this is indispensable for the diagnosis and referral of the case; regular re-evaluations are also required.
- On the other hand, telemedicine may be useful in several circumstances, including the ones below, among others:
  b.1. in the reassessment and monitoring of known patients, to adjust medications, answer simple questions, check for adherence and others.
  b.2. to share information on additional tests, especially when these are normal. The patient does not have to go back to the office just to know that everything is normal; they should lead a normal life and be re-evaluated within a year, and so on.
  b.3. patients in remote regions where there are no medical resources; such people can receive general guidance as in cases of diarrhea, fractures, childbirth, trauma and other ordinary situations. General guidance will be at the discretion of the central physician.
  b.4. to avoid unnecessary hospital visits, such as to get results of simple tests, prothrombin time, in which case medical advice can be given at a distance, saving time and discomfort in addition to reducing costs.
  b.5. to advise on the choice of specialists for specific cases.
  b.6. to reduce hospitalization time - this is perfectly possible as long as the patient is monitored after discharge.
  b.7. in cases where there is a long waiting for a visit, as in public healthcare, follow-up by telemedicine may facilitate or redirect the case.

In short, any innovation can bring progress and also new challenges. With telemedicine, it is not different. The concept, however, needs to be well understood. Telemedicine has not come to fully replace traditional practice. It is here to perfect it. The doctor’s responsibility remains the same; the doctor is the one who will make the main decisions.

We should use the best of the two worlds: preserving humanism in medicine and using new technologies to improve medical care. This is possible; it only depends on some adjustments that are evidently possible. However, further studies are still needed to answer important questions such as those mentioned above. These questions include patient acceptance, the effectiveness of telemedicine in specific clinical conditions and the impact on the health system as a whole. One possibility that cannot be ruled out is that telemedicine and the telecommunication media may become so efficient in the future, to the point that doctor/patient relationships are drastically changed, and what is causing concern today is outweighed by other much more significant advantages. This has happened to iPhones and the social media: no one writes letters anymore. And no one misses them.

However, while cost reduction in the health system is an important element, on the other, cost reduction alone cannot be the only standard of analysis for a new technology such as telemedicine. It is always important to be aware of the benefits for the patients.
